# Supplementary material for: Photothermal-enabled single-atom catalysts for high-efficiency hydrogen peroxide photosynthesis from natural seawater
Source: Nat Commun. 2023 Apr 29;14:2493. doi: 10.1038/s41467-023-38211-3 (PMC10148870; doi:10.1038/s41467-023-38211-3)
Supplement: Supplementary file 1 — Supplementary Information [file 41467_2023_38211_MOESM1_ESM.pdf]

Supplementary Information for

Photothermal-enabled single-atom catalysts for high-efficiency hydrogen peroxide photosynthesis from natural seawater

Wei Wang[1,2†], Qun Song[3†], Qiang Luo[1*], Linqian Li[1], Xiaobing Huo[1], Shipeng Chen[1], Jinyang Li[1], Yunhong Li[1], Se Shi[1], Yihui Yuan[1], Xiwen Du[2], Kai Zhang[3], Ning Wang[1*]

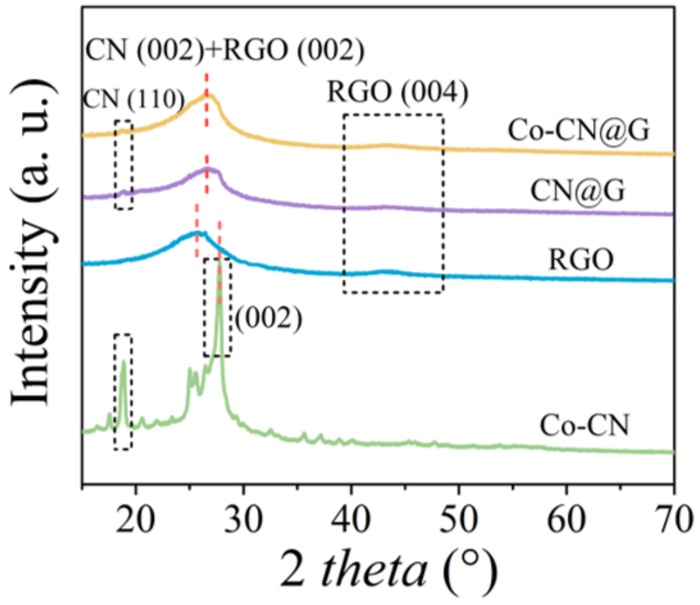

Supplementary Figure 1. XRD patterns of Co-CN@G, CN@G, RGO, and Co-CN.

Source data are provided as a Source Data file.

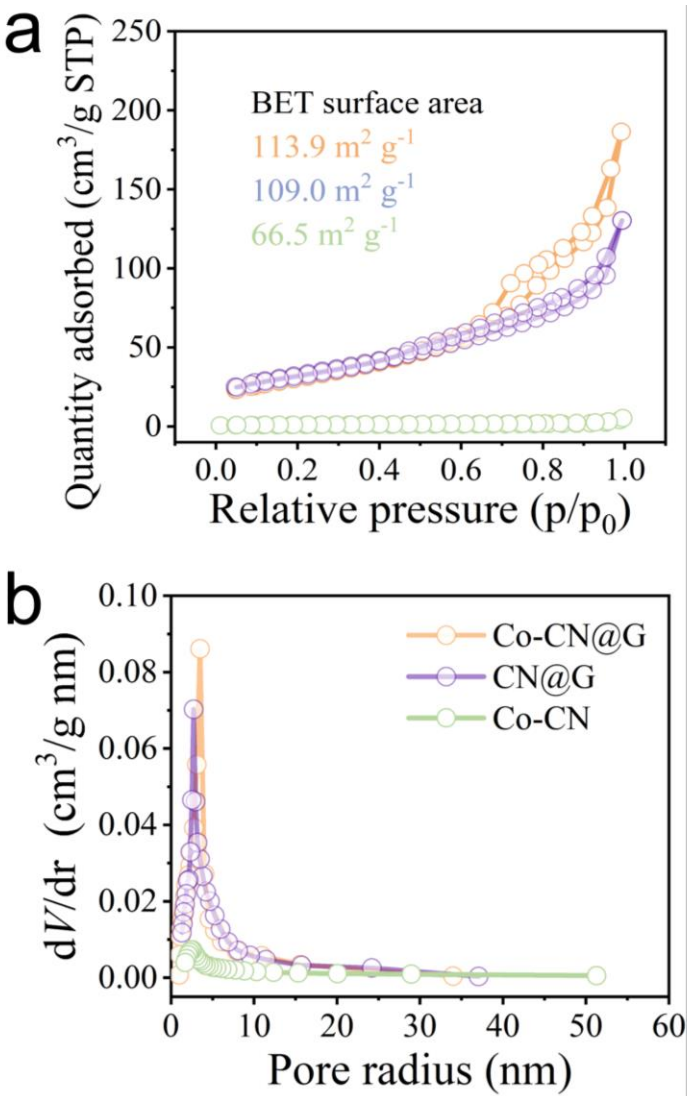

Supplementary Figure 2. a) Nitrogen adsorption-desorption isotherms of Co-CN@G, CN@G, and Co-CN. b) The corresponding pore size distributions. Source data are provided as a Source Data file.

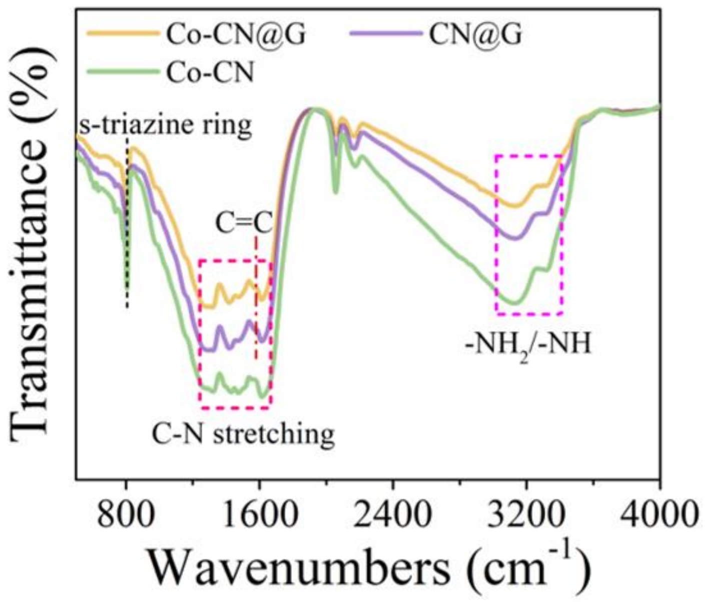

Supplementary Figure 3. FT-IR spectrum of Co-CN@G, CN@G, and Co-CN.

Source data are provided as a Source Data file.

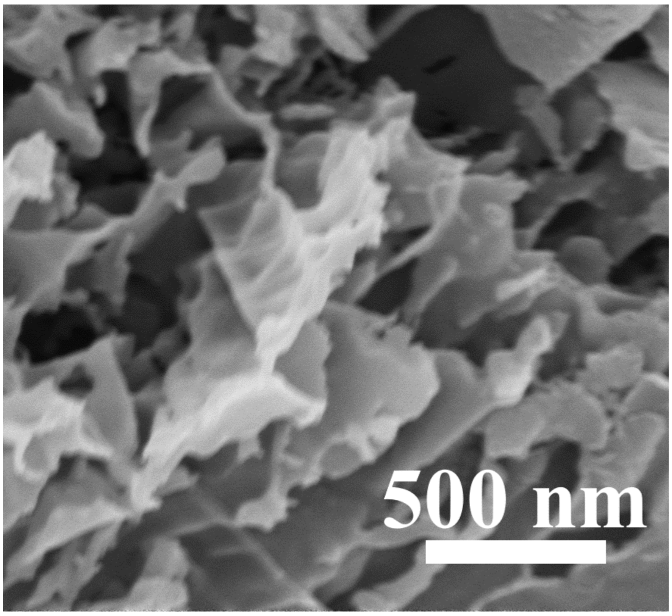

Supplementary Figure 4. SEM image of Co-CN@G.

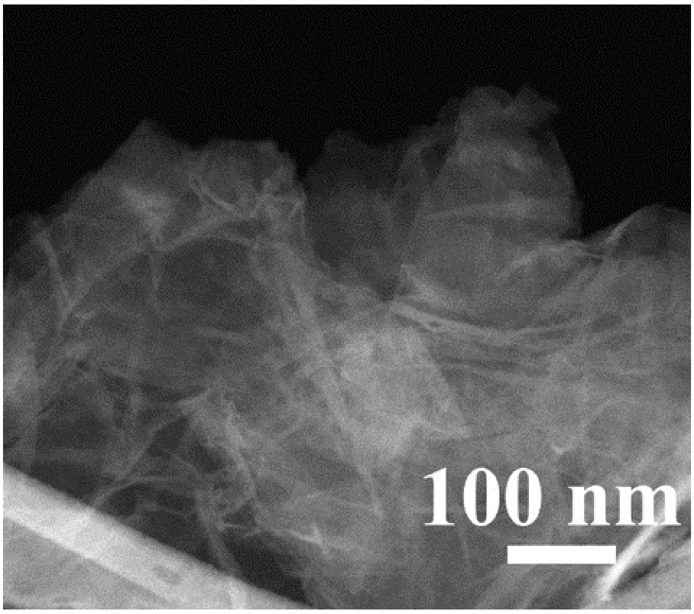

Supplementary Figure 5. HADDF-STEM image of Co-CN@G.

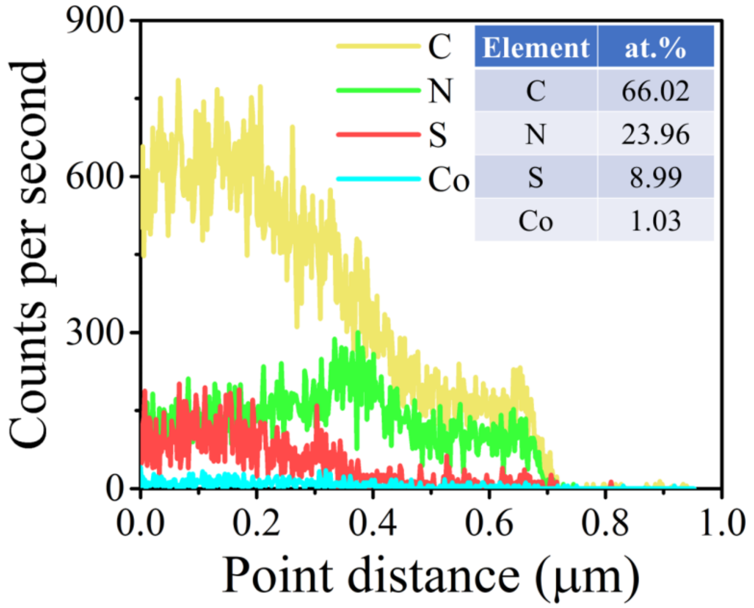

Supplementary Figure 6. TEM-EDS pattern of Co-CN@G. Source data are provided as a Source Data file.

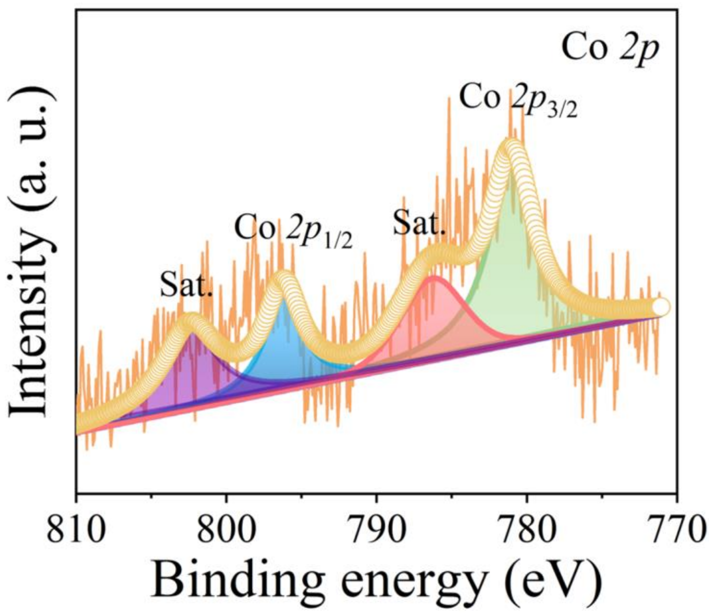

Supplementary Figure 7. High-resolution XPS spectra of Co *2p*. Source data are provided as a Source Data file.

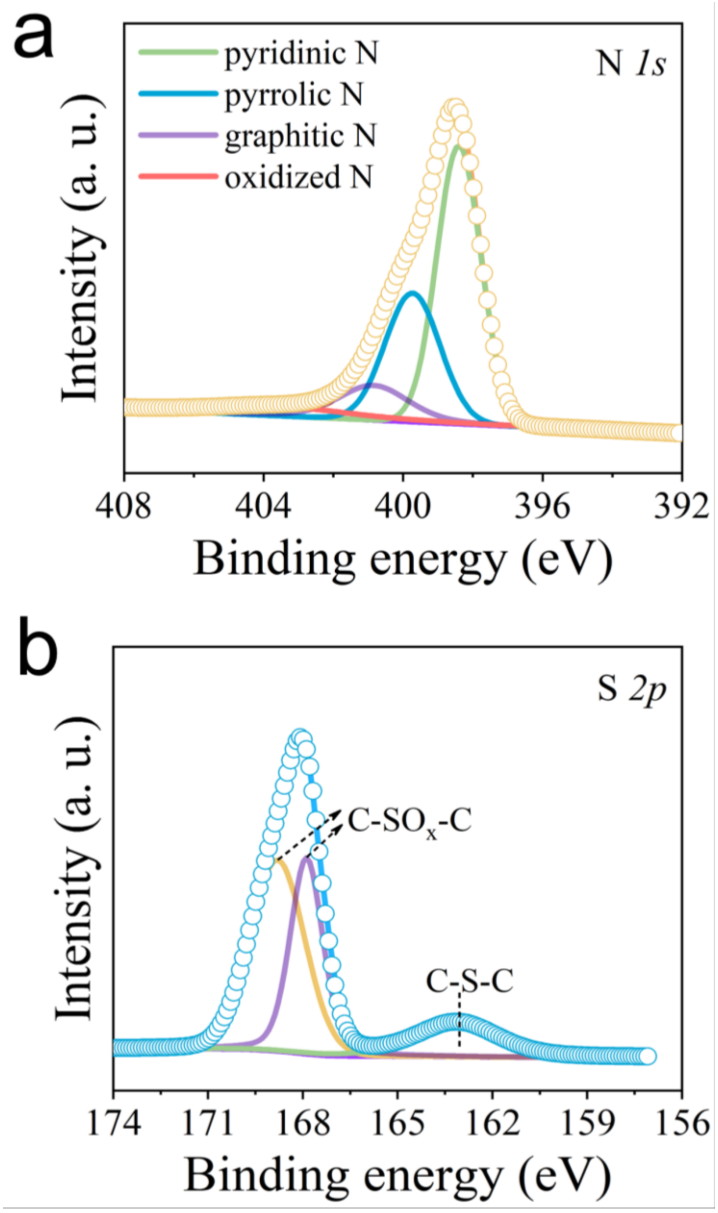

Supplementary Figure 8. High-resolution XPS spectra of a) N *1s* and b) S *2p*.

Source data are provided as a Source Data file.

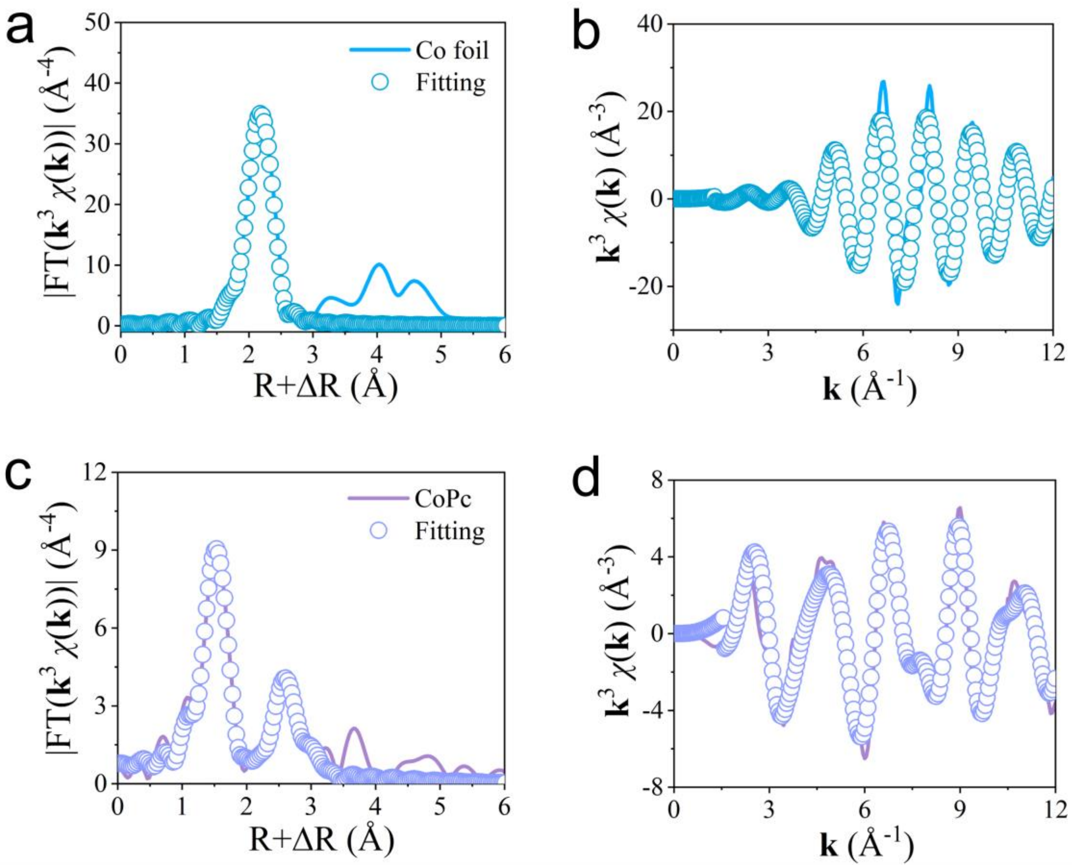

Supplementary Figure 9. The EXAFS fitting curves of a, b) Co foil, and c, d) CoPc for Co K-edge at R and k space, respectively. Source data are provided as a Source Data file.

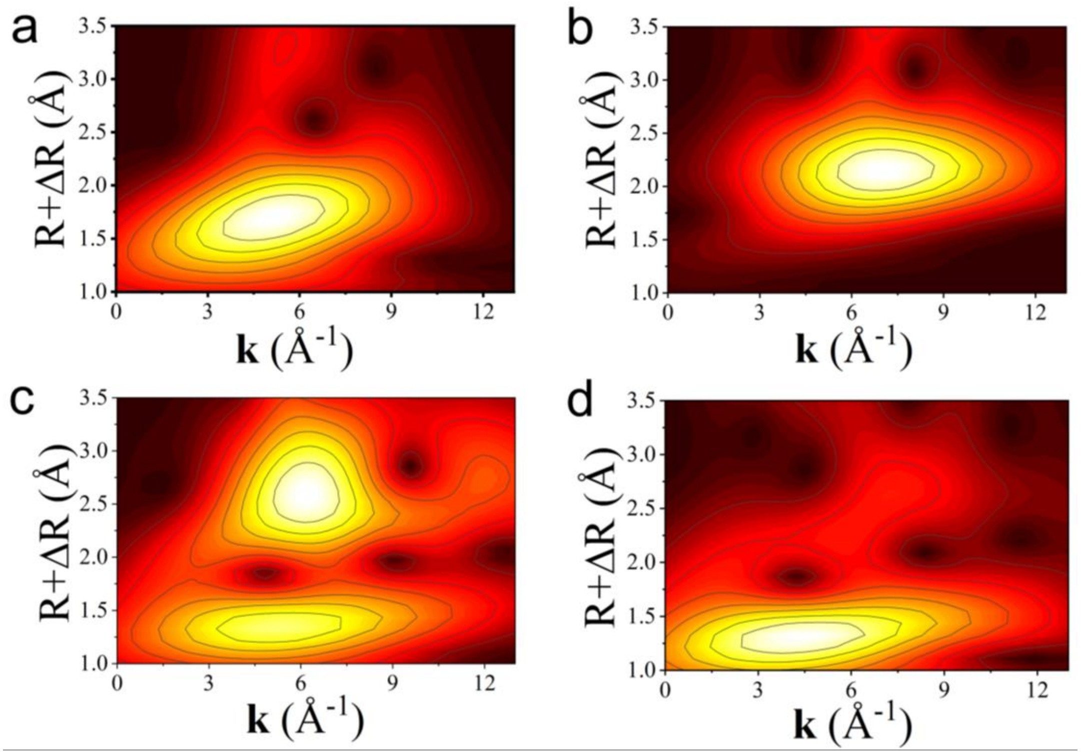

Supplementary Figure 10. WT-EXAFS of a) Co-CN@G, b) Co foil, c) Co_3O_4, and d) CoPc. Source data are provided as a Source Data file.

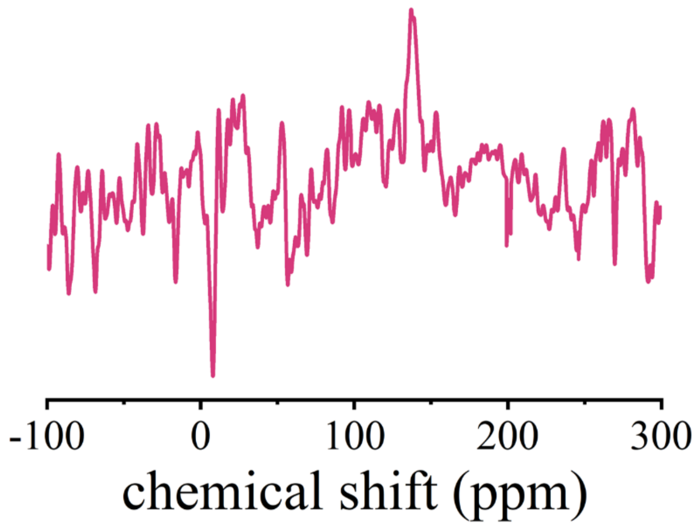

Supplementary Figure 11. ^{15}N solid-state NMR MAS spectra of Co-CN@G. Source data are provided as a Source Data file.

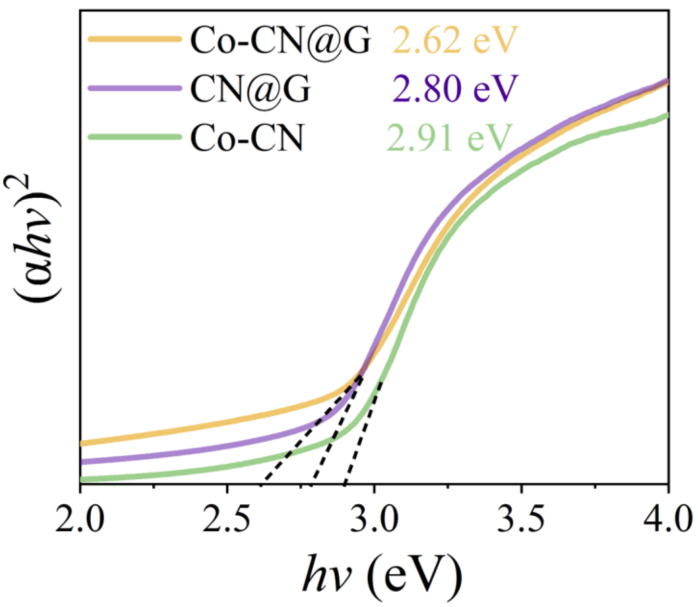

Supplementary Figure 12. The transformed Kubelka-Munk function plots versus photon energy of Co-CN@G, CN@G, and Co-CN. Source data are provided as a Source Data file.

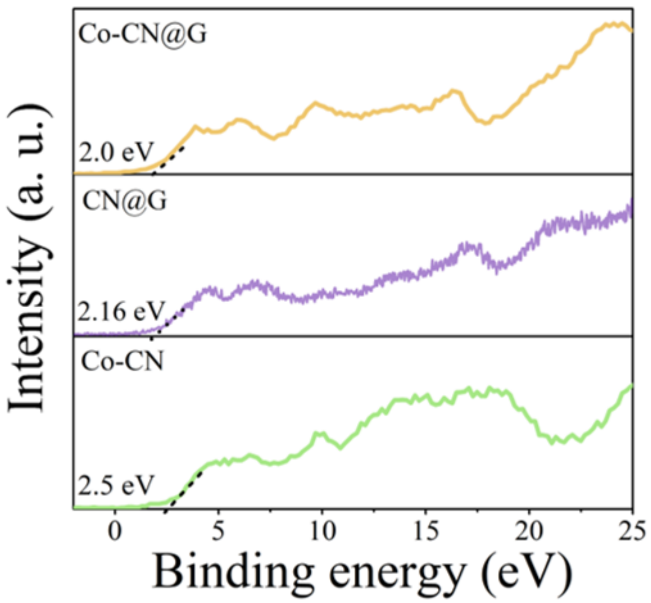

Supplementary Figure 13. XPS valence band spectra of Co-CN@G, CN@G, and Co-CN. Source data are provided as a Source Data file.

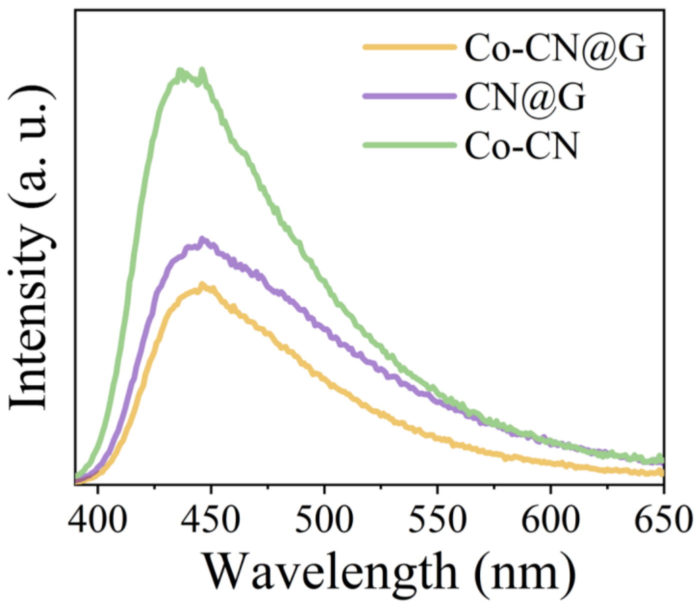

Supplementary Figure 14. Steady-state PL emission spectra (excitation at 375 nm) of Co-CN@G, CN@G, and Co-CN. Source data are provided as a Source Data file.

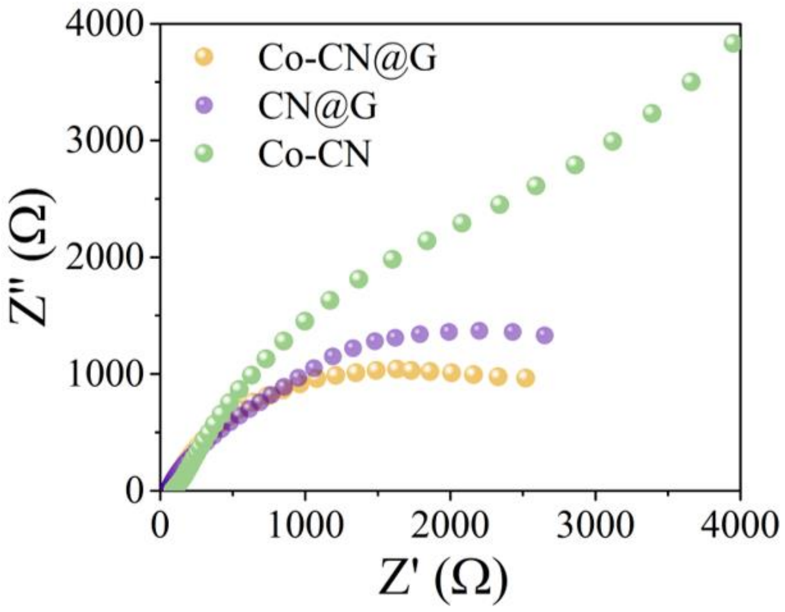

Supplementary Figure 15. Electrochemical impedance spectroscopy Nyquist plots of Co-CN@G, CN@G, and Co-CN electrodes. Source data are provided as a Source Data file.

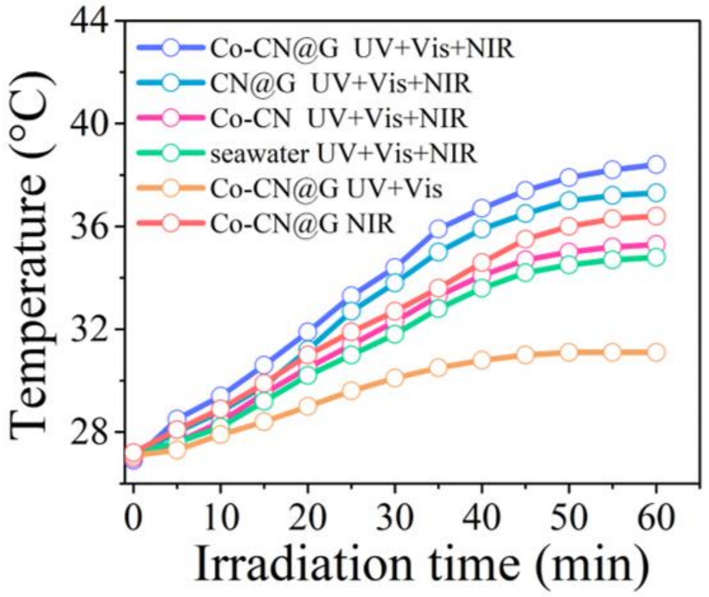

Supplementary Figure 16. Temperature variation determined from seawater, Co-CN@G, CN@G, and Co-CN suspensions under different photoirradiation. Source data are provided as a Source Data file.

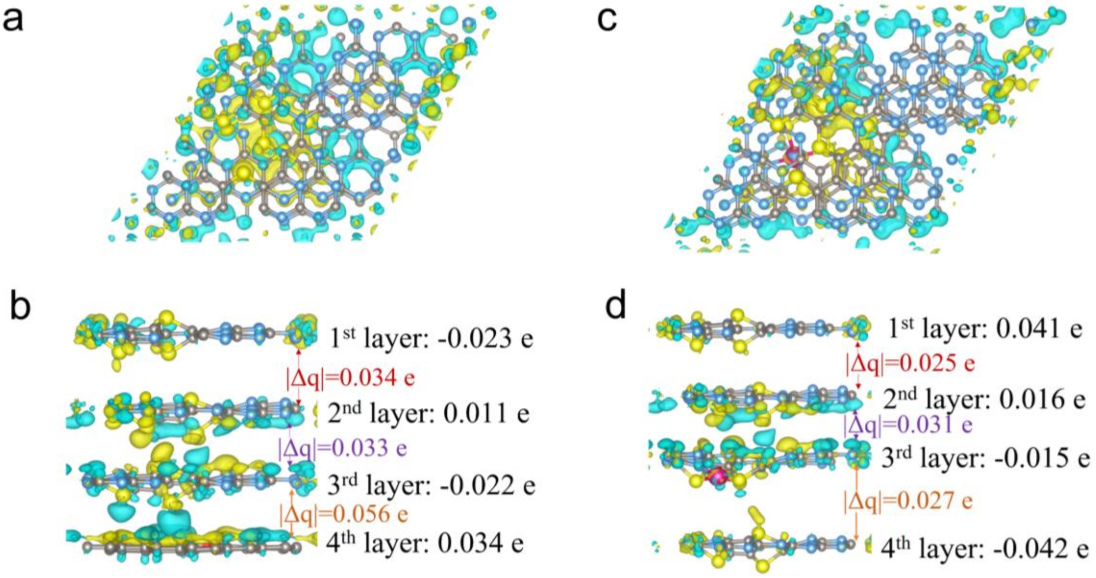

Supplementary Figure 17. The Mulliken charge difference between each adjacent layers of a) Enlarged top view, b) cross view of CN@G. c) Enlarged top view, and d) cross view of Co-CN. Yellow and cyan iso-surface represents electron accumulation and electron depletion, respectively. Blue, gray, yellow, rose, and red color represent nitrogen, carbon, sulphur, cobalt, and oxygen, respectively.

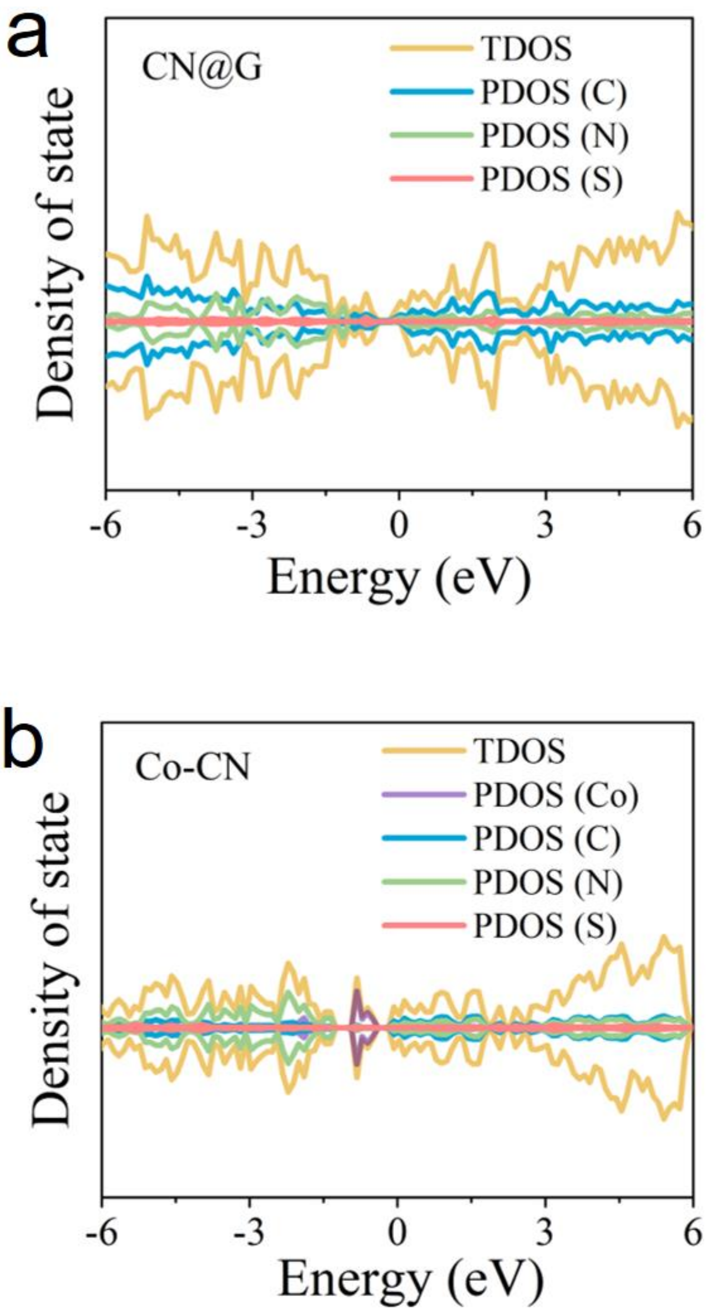

Supplementary Figure 18. The calculated total density of states (TDOS) and partial density of states (PDOS) of a) CN@G, and b) Co-CN. Source data are provided as a Source Data file.

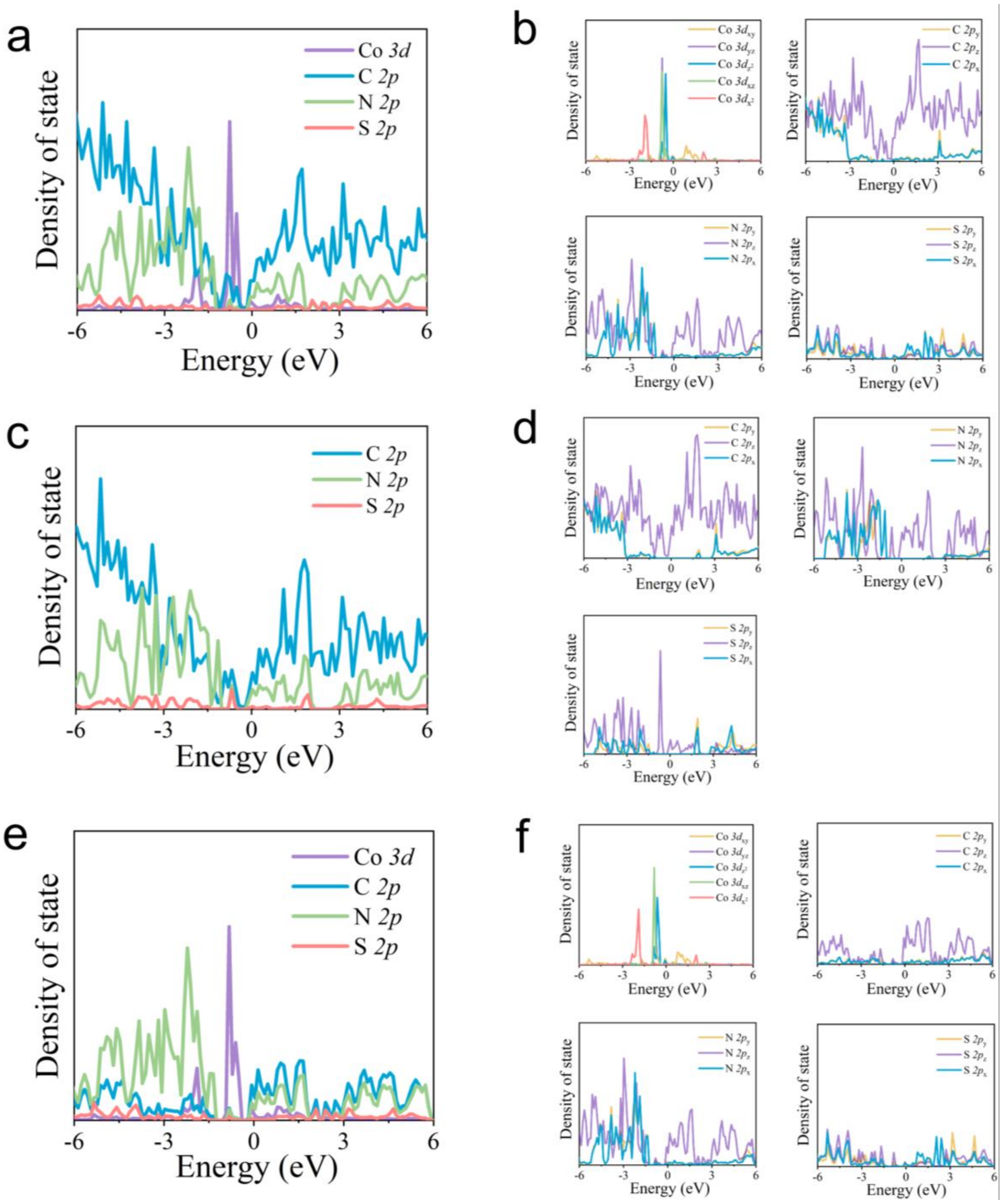

Supplementary Figure 19. The calculated projected density of states (PDOS) of a, b) Co-CN@G, c, d) CN@G, and e, f) Co-CN. Source data are provided as a Source Data file.

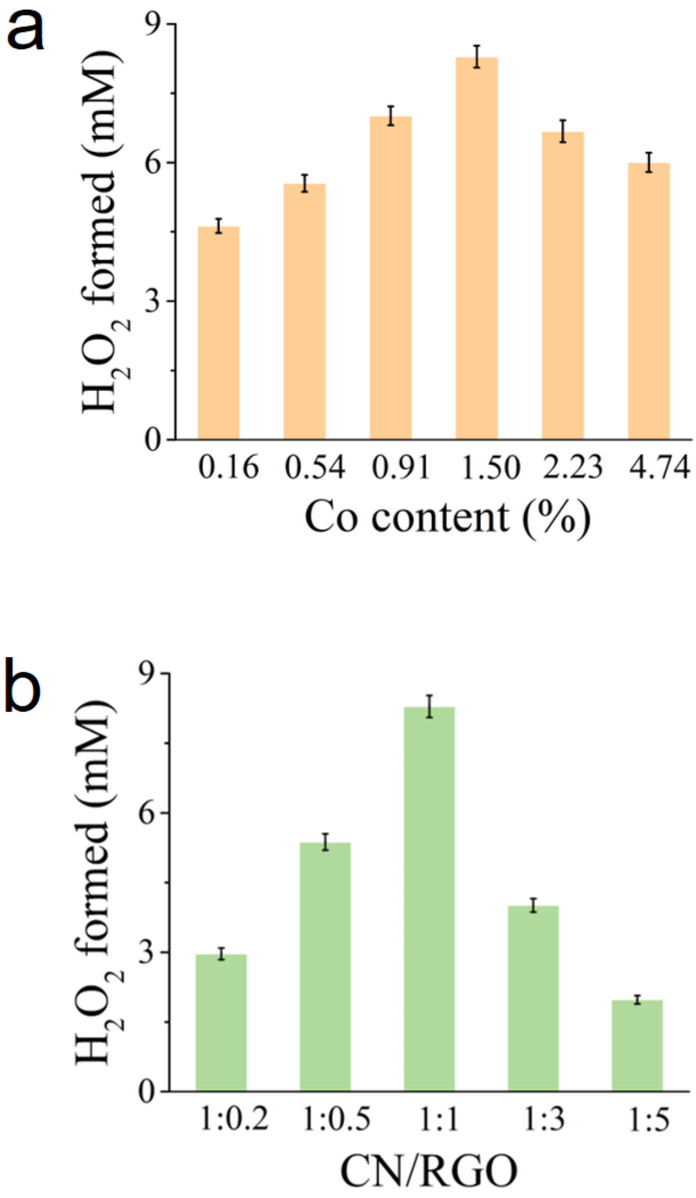

Supplementary Figure 20. Effect of a) Co loading, and b) mass ratio of component on the H_2O_2 photoproduction. Error bars indicate standard deviation for three measurements. Source data are provided as a Source Data file.

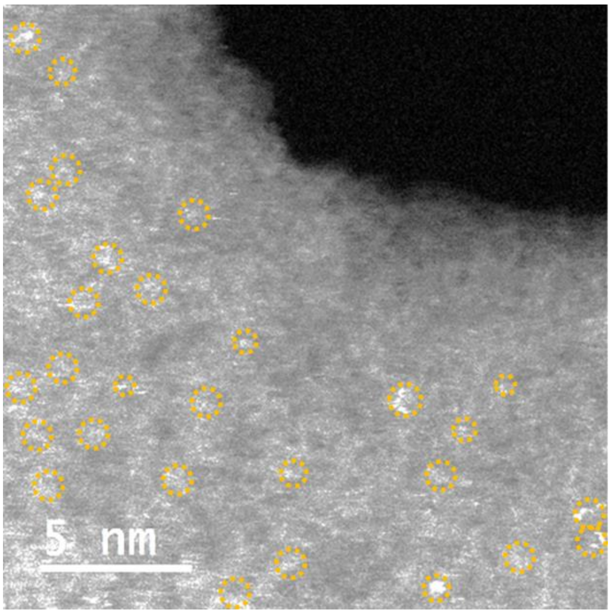

Supplementary Figure 21. HADDF-STEM image of Co-CN@G with 2.8 wt.% Co loading. The bright spots of Co nanoclusters and particles are marked with yellow circles.

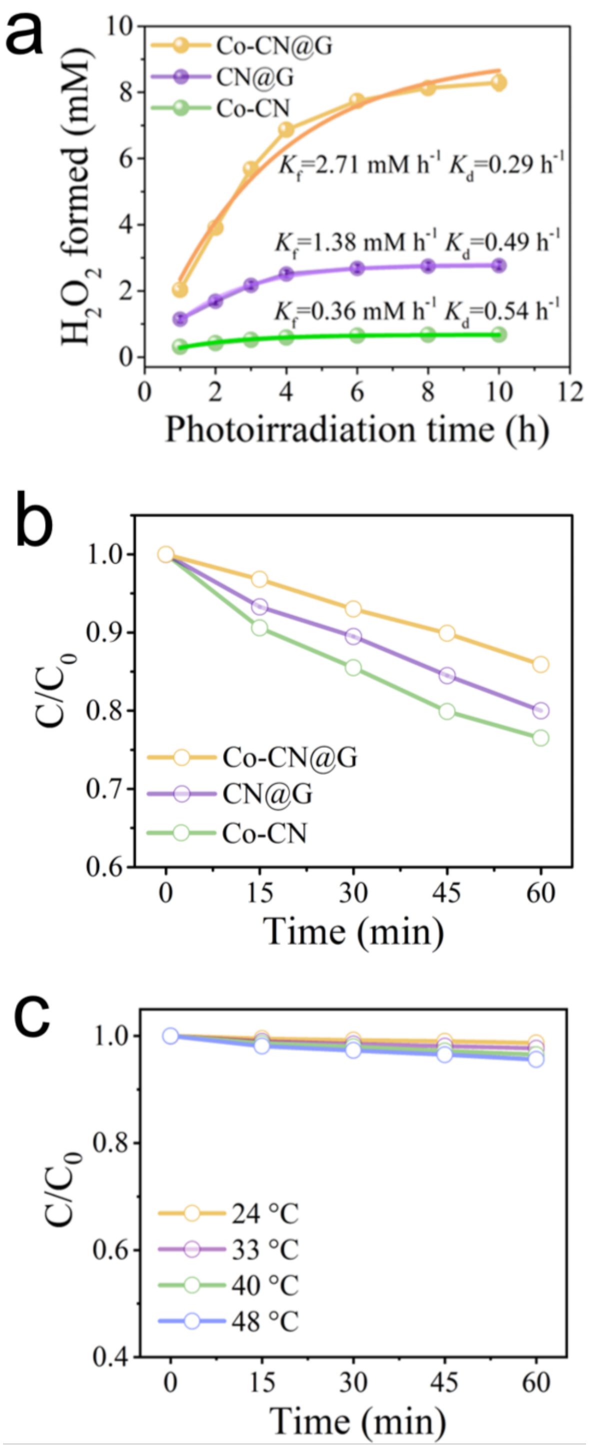

Supplementary Figure 22. a) Time course of H_2O_2 photoproduction by Co-CN@G, CN@G, and Co-CN. b) The photocatalytic decomposition of H_2O_2 (C_0 = 1 mM) over Co-CN@G, CN@G, and Co-CN samples. c) The decomposition of H_2O_2 at different temperatures in the presence of Co-CN@G under dark. Error bars indicate standard deviation for three measurements. Source data are provided as a Source Data file.

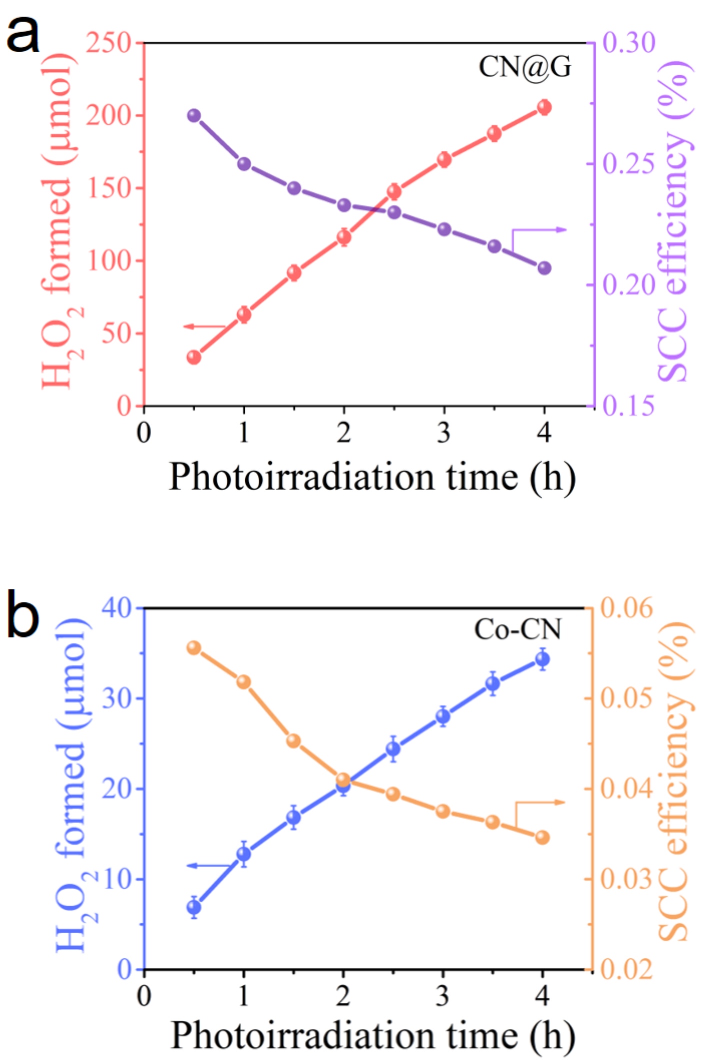

Supplementary Figure 23. Changes in the amounts of H_2O_2 generated on a) CN@G, and b) Co-CN and the solar-to-chemical (SCC) efficiency under AM1.5G simulated sunlight irradiation. Error bars indicate standard deviation for three measurements. Source data are provided as a Source Data file.

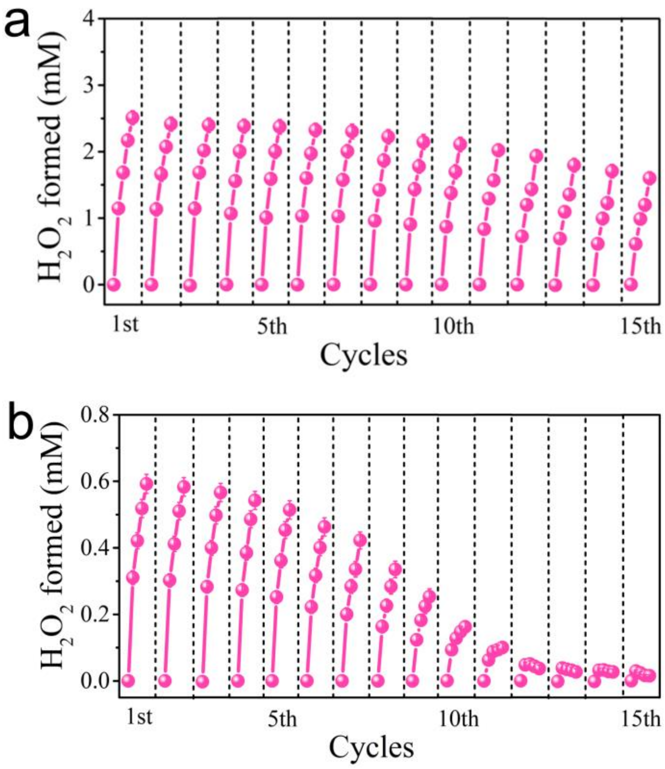

Supplementary Figure 24. The stability of photocatalysts after continuous photocatalytic reactions: a) CN@G, and b) Co-CN. Error bars indicate standard deviation for three measurements. Source data are provided as a Source Data file.

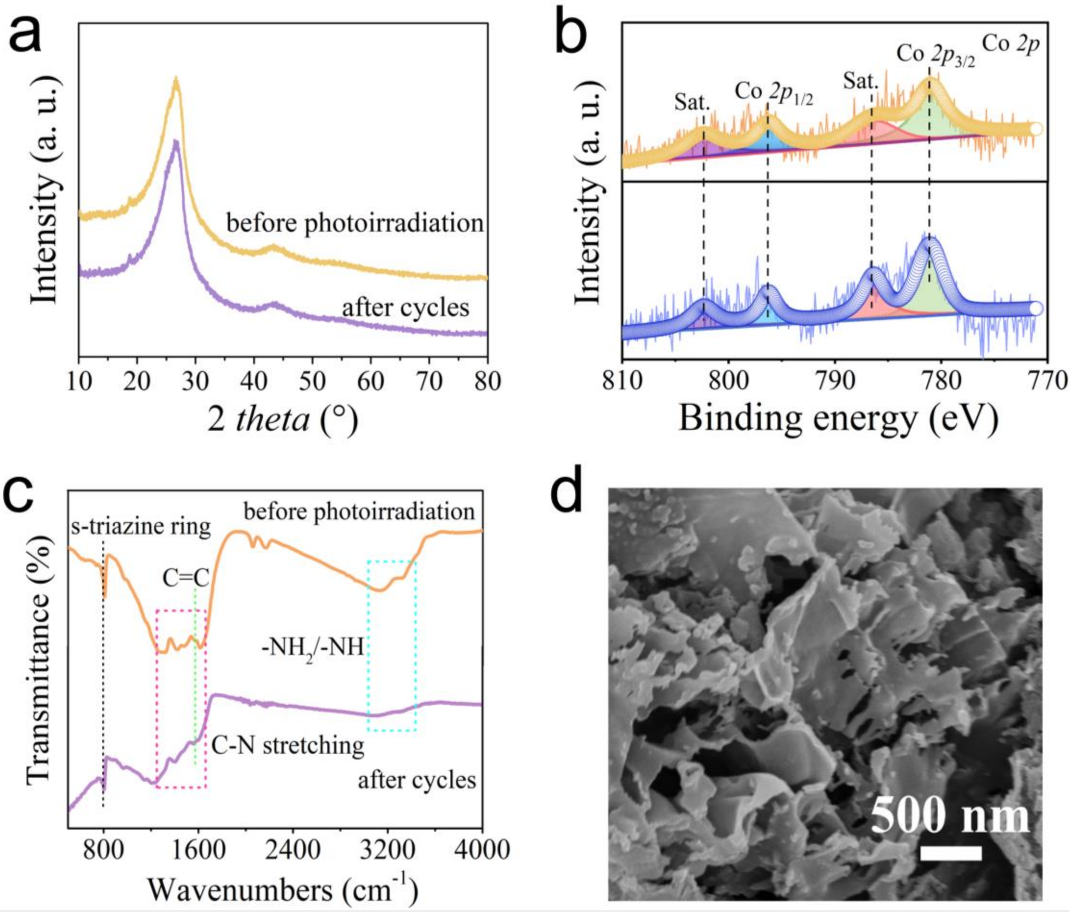

Supplementary Figure 25. a) XRD pattern, b) High-resolution XPS spectra of Co *2p*, c) FT-IR spectrum, and d) SEM image of Co-CN@G after 15 cycles of photoirradiation. Source data are provided as a Source Data file.

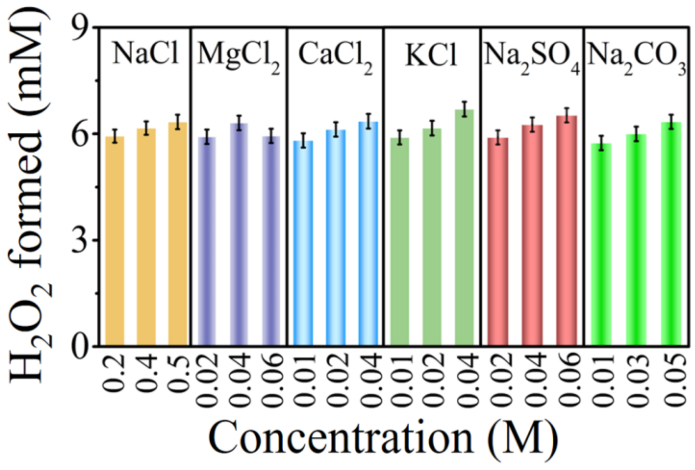

Supplementary Figure 26. Photocatalytic H_2O_2 production (6 h) using the Co-CN@G catalyst in water with various additives. Error bars indicate standard deviation for three measurements. Source data are provided as a Source Data file.

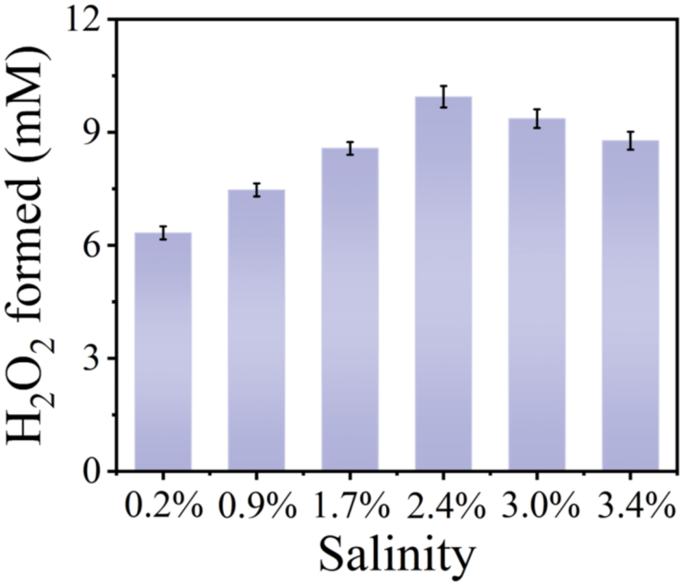

Supplementary Figure 27. Photocatalytic H_2O_2 synthesis from simulated seawater (10 h) with different salinities. Error bars indicate standard deviation for three measurements. Source data are provided as a Source Data file.

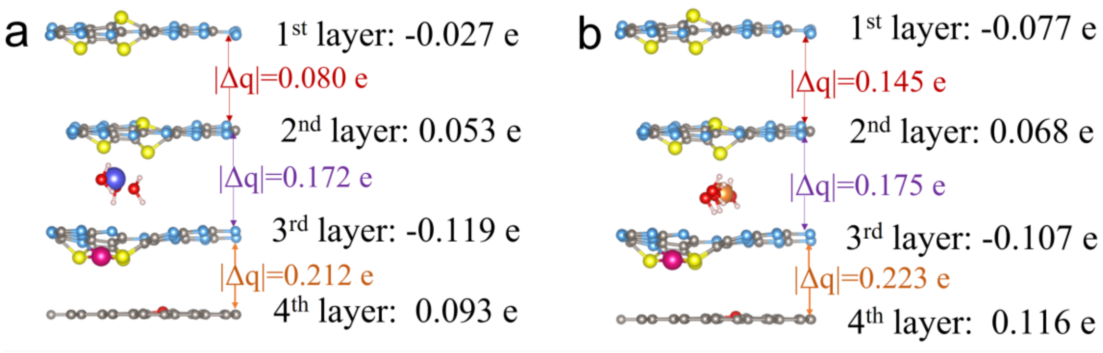

Supplementary Figure 28. The Mulliken charge difference between each adjacent layers of Co-CN@G for the enlarged cross view in the presence of a) $Na^{+}(H_2O)_3$, and b) $Mg^{2+}(H_2O)_4$. Blue, gray, yellow, rose, red, light pink, purple, and orange color represent nitrogen, carbon, sulphur, cobalt, oxygen, hydrogen, sodium, and magnesium, respectively.

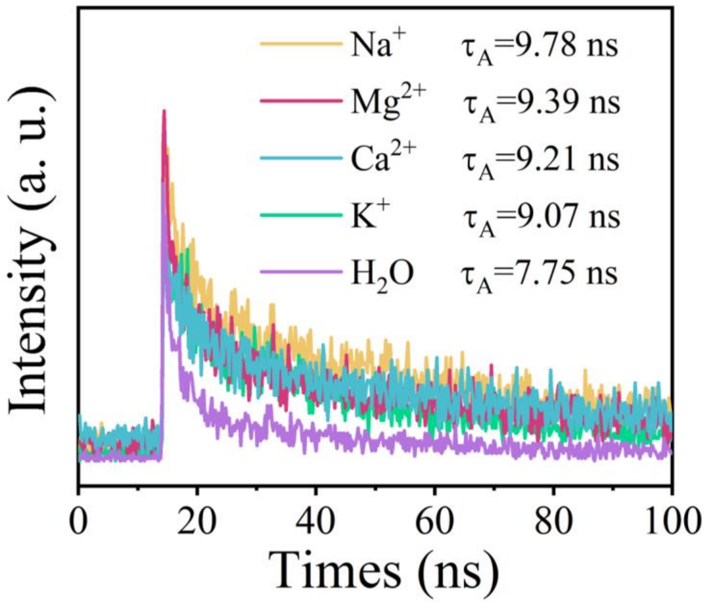

Supplementary Figure 29. Time-resolved PL spectra of Co-CN@G in different metal chlorides solutions (0.05 M). Source data are provided as a Source Data file.

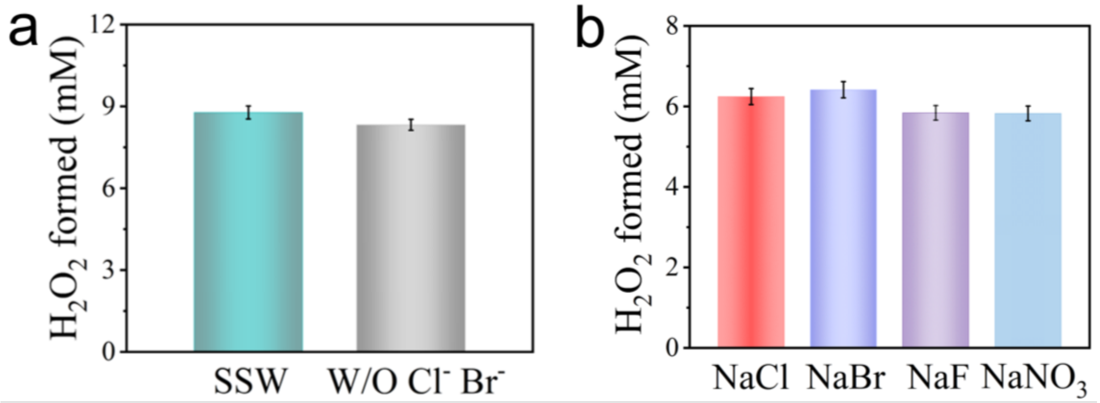

Supplementary Figure 30. a) Photocatalytic H_2O_2 synthesis from simulated seawater (SSW) for 10 h in the absence of (W/O) Cl^- and Br^-. b) H_2O_2 photoproduction in different solutions (0.05 M) for 10 h. Error bars indicate standard deviation for three measurements. Source data are provided as a Source Data file.

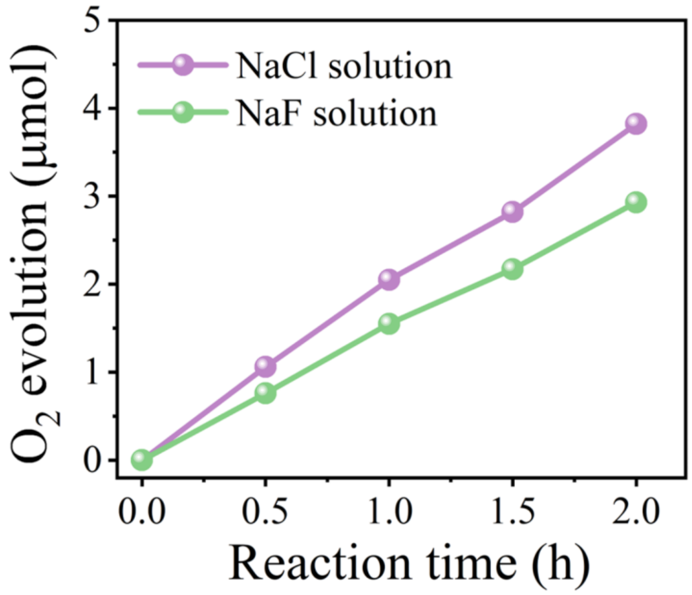

Supplementary Figure 31. Time course of photocatalytic O_2 evolution over Co-CN@G in NaCl and NaF solution (0.05 M) during the half reaction. Source data are provided as a Source Data file.

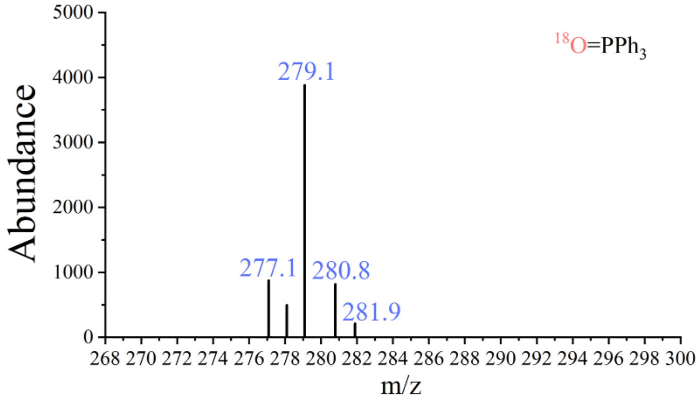

Supplementary Figure 32. GC-MS spectrum of the $^{18}O_2$ labelled reaction using triphenylphosphine as the capping agent. Source data are provided as a Source Data file.

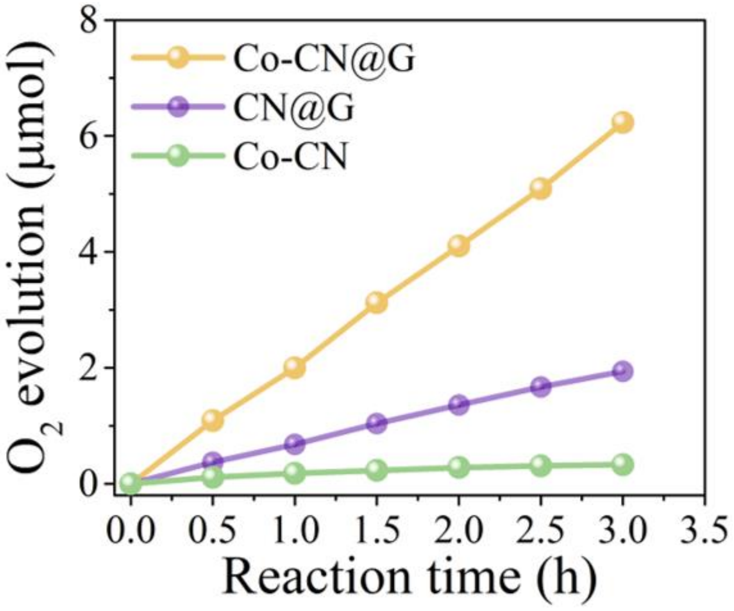

Supplementary Figure 33. Time course of photocatalytic O_2 evolution measured over Co-CN@G, CN@G, and Co-CN during the half reaction. Source data are provided as a Source Data file.

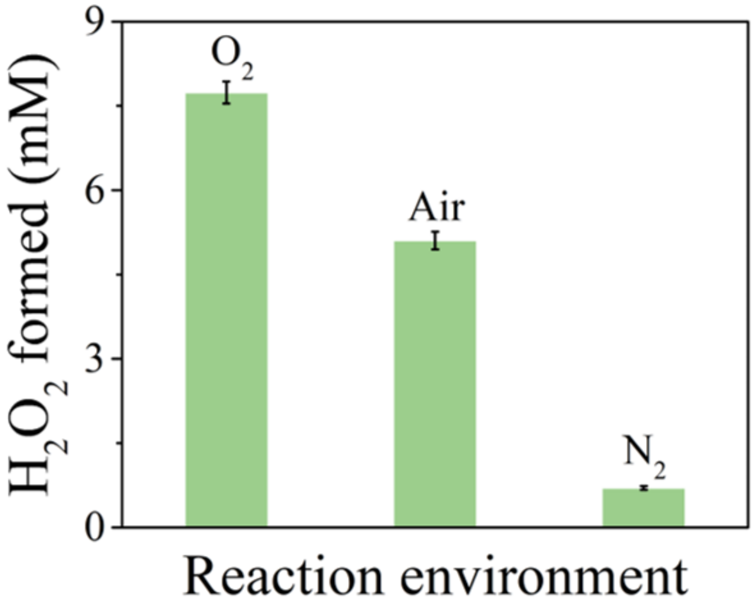

Supplementary Figure 34. The production of H_2O_2 by photocatalytic reaction in different gas environments. Error bars indicate standard deviation for three measurements. Source data are provided as a Source Data file.

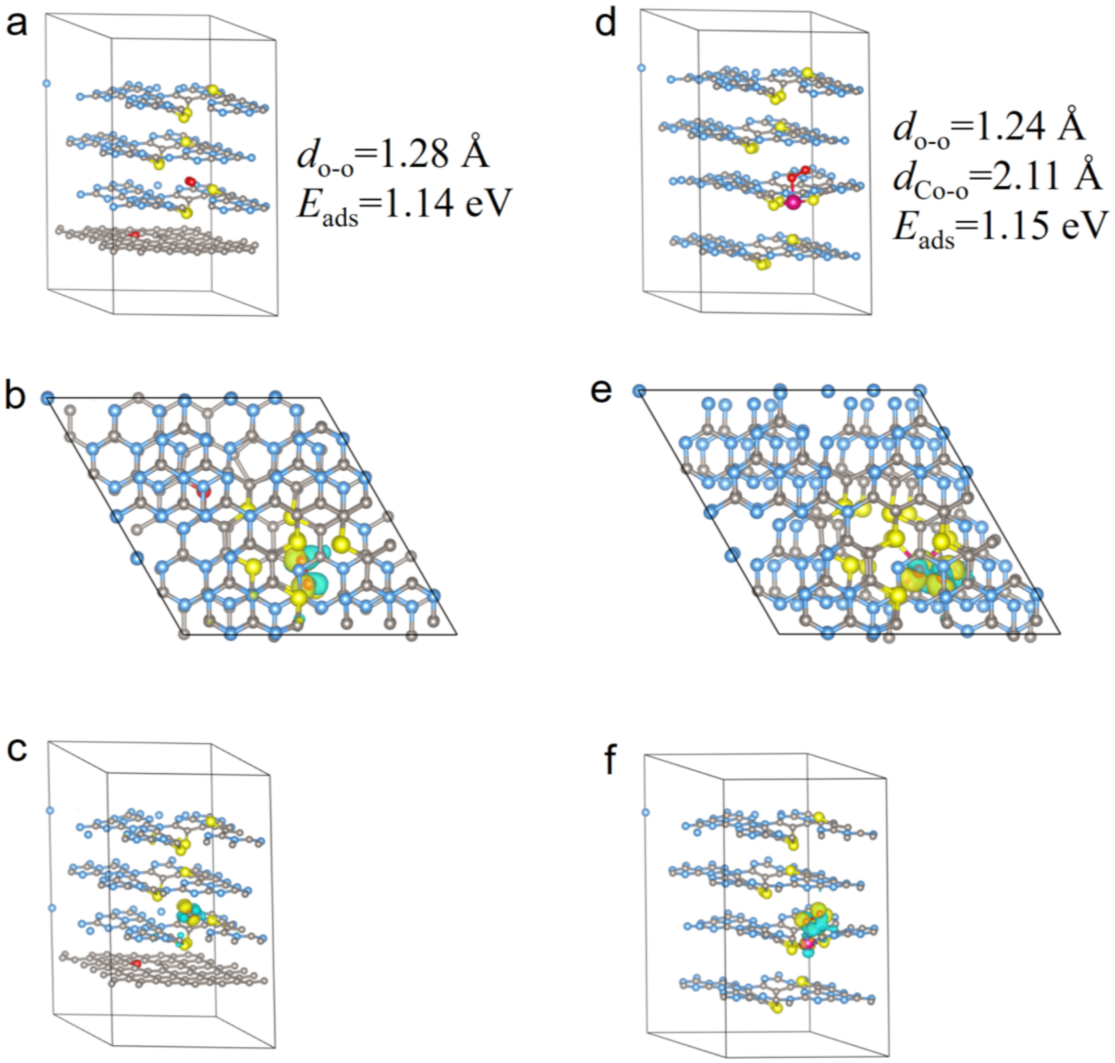

Supplementary Figure 35. a, d) Cross view for O_2 adsorption configuration of CN@G and Co-CN. The charge difference density of O_2 adsorption for the enlarged b) top view, c) cross view for CN@G, and e, f) for Co-CN, respectively. Yellow and cyan iso-surface represents electron accumulation and electron depletion, respectively. Blue, gray, yellow, rose, and red color represent nitrogen, carbon, sulphur, cobalt, and oxygen, respectively.

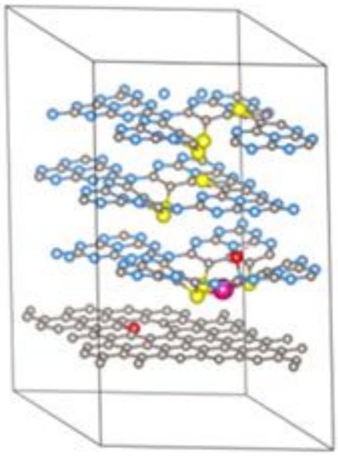

Supplementary Figure 36. The most stable configuration of reaction intermediates OH* on the N site of Co-CN@G. Blue, gray, yellow, rose, and red color represent nitrogen, carbon, sulphur, cobalt, and oxygen, respectively.

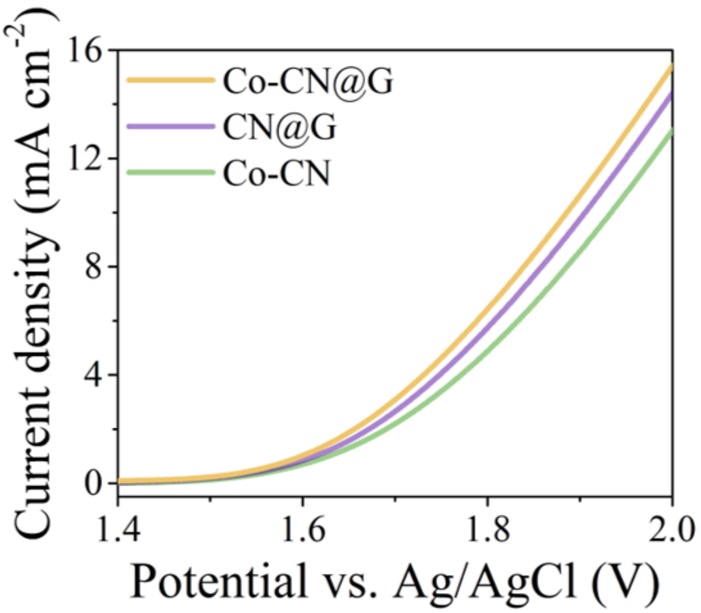

Supplementary Figure 37. LSV of Co-CN@G, CN@G, and Co-CN measured on carbon paper with scan rate of 10 mV s^{-1}. Source data are provided as a Source Data file.

Supplementary Table 1. EXAFS fitting parameters at the Co K-edge for various samples (S_0^2=0.899).

Sample	Shell	CN	R (Å)	σ^2 ($Å^2$)	ΔE_0 (eV)	R factor
Co foil	Co-Co	12*	2.489±0.002	0.0064±0.0003	6.9±0.4	0.0033
CoPc	Co-N	4.0±0.2	1.915±0.005	0.0026±0.0007	4.2±1.3	0.0078
	Co-C	6.0±0.5	2.937±0.010	0.0021±0.0011		
Co-CN@G	Co-N	0.9±0.3	1.945±0.016	0.0027±0.0012	2.8±2.4	0.0119
	Co-S	2.8±0.6	2.293±0.019			

CN, coordination number; R, the distance to the neighboring atom; σ^2, the Mean Square Relative Displacement (MSRD); ΔE_0, inner potential correction; R factor indicates the goodness of the fit. S_0^2 was fixed to 0.899, according to the experimental EXAFS fit of Co foil by fixing CN as the known crystallographic value. * This value was fixed during EXAFS fitting based on the known structure of Co foil. Fitting range: $3.0 \le k$ (/Å) ≤ 14.0 and $1.0 \le R$ (Å) ≤ 3.0 (Co foil); $3.0 \le k$ (/Å) ≤ 12.6 and $1.0 \le R$ (Å) ≤ 3.0 (CoPc); $2.0 \le k$ (/Å) ≤ 10.5 and $1.0 \le R$ (Å) ≤ 2.5 (Co-CN@G).

Supplementary Table 2. Summary of reported SCC efficiencies for photocatalytic H_2O_2 production without the assistance of sacrificial reagents in pure water or seawater.

Catalysts	Experimental conditions	SCC (%)	H_2O_2 formed (μmol) (amount of catalysts)	Refs
RF-base resins	Water, AM1.5G solar simulator, λ>420 nm, 100 mW cm^{-2}, 333 K.	0.5	260 (250 mg)	1
Sb-SAPC15	Water, AM 1.5G solar simulator, λ>420 nm, 100 mW cm^{-2}	0.61	940 (500 mg)	2
Nv-C≡N-CN	Water, AM 1.5G solar simulator, λ ≥ 420 nm, 100 mW cm^{-2}	0.23	7.077 (20 mg)	3
PEI/C_3N_4	Water, Xenon light equipped with AM 1.5 A filter, UV-Vis-NIR, 100 mW cm^{-2}	0.045	23.08 (100 mg)	4
g-C_3N_4/PDI/rGO	Water, AM 1.5G solar simulator, λ>420 nm, 100 mW cm^{-2}	0.2	38.5 (250 mg)	5
CTF-BDDBN	Water, AM 1.5G solar simulator, λ>420 nm, 100 mW cm^{-2}	0.14	633.6 (100 mg)	6
PM-CDs-30	Seawater, multichannel photochemical reaction system, λ≥420 nm, 34.8 mW cm^{-2}	0.21	88.8 (10 mg)	7
Lignin/BiOBr	Seawater, 40 W Kessil LED lamp, λ =427 nm	/	134.8 (45 mg)	8
Co-CN@G	Seawater, AM 1.5G solar simulator, UV-Vis-NIR, 100 mW cm^{-2}	0.72	707.8 (200 mg)	This work

Supplementary Table 3. Simulated seawater without one or two ions for photocatalytic reactions. The following chemicals are dissolved in 1L freshwater.

Removed ion	Dissolved chemicals
Na^{+}	NH_4Cl, 24.90 g; $MgCl_2$, 3.81 g; $MgSO_4$, 1.66 g; $CaSO_4$, 1.40 g; K_2SO_4, 0.58 g; K_2CO_3, 0.21 g; $MgBr_2$, 0.08 g
K^{+}	NaCl, 27.21 g; $MgCl_2$, 3.81 g; $MgSO_4$, 1.66 g; $CaSO_4$, 1.40 g; $(NH_4)_2SO_4$, 0.64 g; $MgBr_2$, 0.08 g
Mg^{2+}	NaCl, 27.21 g; NH_4Cl, 4.28 g; $(NH_4)_2SO_4$, 1.82 g; $CaSO_4$, 1.40 g; K_2SO_4, 0.58 g; K_2CO_3, 0.21 g; NH_4Br, 0.085 g
Ca^{2+}	NaCl, 27.21 g; $MgCl_2$, 3.81 g; $MgSO_4$, 1.66 g; $(NH_4)_2SO_4$, 1.36 g; K_2SO_4, 0.58 g; K_2CO_3, 0.21 g; $MgBr_2$, 0.08 g
Cl^{-}, Br^{-}	NaF, 19.55 g; $Mg(NO_3)_2 \cdot 6H_2O$, 10.37 g; $MgSO_4$, 1.66 g; $CaSO_4$, 1.40 g; K_2SO_4, 0.58 g; K_2CO_3, 0.21 g;

Supplementary References

1. Shiraishi, Y. et al. Resorcinol-formaldehyde resins as metal-free semiconductor photocatalysts for solar-to hydrogen peroxide energy conversion. *Nat. Mater.* **18**, 985-993 (2019).
2. Teng, Z. et al. Atomically dispersed antimony on carbon nitride for the artificial photosynthesis of hydrogen peroxide. *Nat. Catal.* **4**, 374-384 (2021).
3. Zhang, X. et al. Unraveling the dual defect sites in graphite carbon nitride for ultra-high photocatalytic H_2O_2 evolution. *Energy Environ. Sci.* **15**, 830-842 (2022).
4. Zeng, X. et al. Simultaneously tuning charge separation and oxygen reduction pathway on graphitic carbon nitride by polyethylenimine for boosted photocatalytic hydrogen peroxide production. *ACS Catal.* **10**, 3697-3706 (2020).
5. Kofuji, Y. et al. Carbon nitride-aromatic diimide-graphene nanohybrids: metal-free photocatalysts for solar-to-hydrogen peroxide energy conversion with 0.2% efficiency. *J. Am. Chem. Soc.* **138**, 10019-10025 (2016).
6. Chen, L. et al. Acetylene and diacetylene functionalized covalent triazine frameworks as metal-free photocatalysts for hydrogen peroxide production: a new two-electron water oxidation pathway. *Adv. Mater.* **32**, 1904433 (2020).
7. Wu, Q. et al. A metal-free photocatalyst for highly efficient hydrogen peroxide photoproduction in real seawater. *Nat. Commun.* **12**, 483 (2021).
8. Gopakumar, A. et al. Lignin-supported heterogeneous photocatalyst for the direct generation of H_2O_2 from seawater. *J. Am. Chem. Soc.* **144**, 2603-2613 (2022).
